# Interannual variability in the lipid and fatty acid profiles of east Australia-migrating humpback whales (*Megaptera novaeangliae*) across a 10-year timeline

**DOI:** 10.1038/s41598-020-75370-5

**Published:** 2020-10-26

**Authors:** Jasmin Groß, Patti Virtue, Peter D. Nichols, Pascale Eisenmann, Courtney A. Waugh, Susan Bengtson Nash

**Affiliations:** 1 Southern Ocean Persistent Organic Pollutants Program, Environmental Futures Research Institute, Griffith University, 170 Kessels Road, Nathan, QLD 4111 Australia; 2 Institute for Marine and Antarctic Studies, University of Tasmania, 20 Castray Esplanade, Hobart, TAS 7004 Australia; 3 CSIRO Oceans and Atmosphere, Castray Esplanade, Hobart, TAS 7004 Australia; 4 Property NSW – Environmental Science Group, Parramatta Square, Parramatta, NSW Australia; 5 Animal Science Programme, Nord University, Universitetsalléen 11, 8026 Bodø, Norway

**Keywords:** Animal physiology, Climate-change ecology, Fatty acids, Ecological modelling, Ecophysiology, Marine biology

## Abstract

Southern hemisphere humpback whales are classified as high-fidelity Antarctic krill consumers and as such are vulnerable to variability and long-term changes in krill biomass. Evidence of heterogeneous feeding patterns of east coast of Australia migrating humpback whales has been observed, warranting a comprehensive assessment of interannual variability in their diet. We examined the lipid and fatty acid profiles of individuals of the east coast of Australia migrating stock sampled between 2008 and 2018. The use of live-sampled blubber biopsies showed that fatty acid profiles varied significantly among all years. The two trophic indicator fatty acids for Antarctic krill, 20:5ω3 and 22:6ω3 remained largely unchanged across the 10-year period, suggesting that Antarctic krill is the principal prey item. A distance-based linear model showed that 33% of the total variation in fatty acid profiles was explained by environmental variables and climate indices. Most of the variation was explained by the Southern Annular Mode (23.7%). The high degree of variability observed in this study was unexpected for a species that is thought to feed primarily on one prey item. We propose that the observed variability likely arises from changes in the diet of Antarctic krill rather than changes in the whale’s diet.

## Introduction

Under the classical feeding ecology paradigm, southern hemisphere humpback whales are assumed to be high-fidelity Antarctic krill (*Euphausia superba*) consumers^1^, feeding in Antarctic waters during the austral summer. Intense summer feeding is followed by extended fasting during the whales’ migration to their equatorial breeding grounds, with feeding only being resumed in the Southern Ocean the following summer^2,3^. As capital breeders, humpback whales rely on the accumulated energy reserves for breeding, calving and nursing during the migratory fast. Hence, successful migration and reproduction are assumed to depend on the presence of a high biomass of Antarctic krill. However, departures from the classical feeding paradigm, indicated by feeding along the migration routes^4–7^ and diversified biochemical feeding signals^8,9^ have been observed in several southern hemisphere humpback whale breeding populations in recent years, putting the validity of the paradigm under scrutiny.

Feeding along the migration routes has been observed in four of the seven distinct southern hemisphere humpback whale breeding populations recognised by the International Whaling Commission^10^. Humpback whales migrating along the east coast of Australia belong to the E1 breeding population (hereafter E1 humpback whales)^10^. Individuals from this population have been observed to feed on temperate krill (*Nyctiphanes australis*) and small baitfish during the southward migration to their Antarctic feeding grounds^11^. Contrary to whaling data, which only reported short feeding events lasting minutes to hours, recent observations and satellite tracks of migrating E1 humpback whales have revealed multiple feeding stops lasting days to weeks in several highly productive temperate areas along the migration route^2,12^. These observations show higher food consumption rates during migration than previously assumed under the classical feeding paradigm, which is in accordance with findings based on isotopic signatures of stranded E1 humpback whales, but contrary to findings based on the fatty acid profiles of free-swimming E1 individuals.

A study utilising isotopic signatures of baleen plates from stranded E1 humpback whales found that 59% of sampled whales had isotopic signatures that correspond to the classical feeding paradigm^8^. Occasional supplementary feeding, corresponding to either higher trophic levels in Southern Ocean waters, or on krill and higher trophic levels in temperate waters, was observed in 21% of whales. The remaining 20% of sampled whales appeared to have fed exclusively north of the Antarctic Circumpolar Current, with isotopic signatures of two whales showing no feeding in Antarctica in the years prior to death^8^. The biochemically evidenced departure from the classical feeding paradigm indicated in this study may, however, not be the behaviour of healthy individuals, as baleen whale stranding events are biased towards old, young or sick individuals. This assumption is reinforced by a study utilising blubber fatty acid profiles of free-swimming E1 humpback whales^1^. Based on fatty acid ratios, E1 humpback whales followed the classical feeding paradigm as there was no indication of higher trophic level feeding, or extra-Antarctic supplementary feeding as a consumer-prey relationship between E1 humpback whales and *N. australis* was ruled out^1^. The same study uncovered a clear distinction in the blubber fatty acid composition of northern and southern hemisphere humpback whales, which suggests that diet has a stronger impact on fatty acid composition than species-specific metabolism, as northern hemisphere humpback whales are known to have a broader dietary spectrum than southern hemisphere humpback whales^1^. Finally, a difference in the fatty acid composition of E1 humpback whales compared to Antarctic krill was observed, indicating that species-specific metabolism also impacts blubber fatty acid profiles of E1 humpback whales^1^.

The elevated lipid content of Antarctic krill render the species an excellent energy source for humpback whales^13^. In particular, the essential omega-3 long-chain (≥ C_20_) polyunsaturated fatty acids (ω3 LC-PUFA) of the species are a key requirement for growth and reproduction^14^. The two signature fatty acids of Antarctic krill, eicosapentaenoic acid (EPA, 20:5ω3) and docosahexaenoic acid (DHA, 22:6ω3) are essential fatty acids, which cannot be synthesised de novo by mammals^15^. These two fatty acids, like all other dietary LC-PUFA are deposited directly and unmodified into the adipose tissue of whales^15^. However, whales are capable of slightly modifying fatty acids between ingestion and deposition through elongation, shortening or desaturation of the carbon chain^16^. This is typically restricted to saturated and monounsaturated fatty acids, and often inhibited during fasting and consumption of a high-fat diet such as Antarctic krill^15^. Besides direct deposition and modification, the cumulative fatty acid composition of the whale’s adipose tissue is also the result of endogenously derived fatty acids from de novo synthesis. It remains unknown whether the greatest contributor to the adipose tissue fatty acid composition of E1 humpback whales is direct deposition of fatty acids from diet, modification of fatty acids between absorption and deposition, or de novo synthesis. The difference between the fatty acid composition of E1 humpback whales and Antarctic krill found by Waugh et al.^1^ indicates that direct deposition of fatty acids from diet is not the sole pathway of fatty acid incorporation into the adipose tissue of whales, which precludes direct delineation of the relative influence of diet on the whale’s adipose tissue composition.

The above-outlined, diverse lines of evidence suggest more feeding heterogeneity among E1 humpback whales than assumed under the classical feeding paradigm. This may be an indication that the paradigm has either always been an oversimplification of the feeding ecology of E1 humpback whales or that the feeding ecology is subject to more natural ecosystem variability than previously credited. Alternatively, such newly uncovered heterogeneity may be a sign of present-day changes in the feeding ecology of E1 humpback whales related to climate induced variability of the Antarctic sea-ice ecosystem. The sometimes-contradictory findings between the above-mentioned studies, and the use of differing methodologies, emphasise important research gaps surrounding our understanding of the present-day feeding ecology of E1 humpback whales.

The current study is part of the Humpback Whale Sentinel Program (HWSP), which is a long-term biomonitoring program for circum-polar surveillance of the Antarctic sea-ice ecosystem^9^. It contributes information regarding the sentinel parameters of humpback whale diet, adiposity and fecundity, through quantification of chemical and biochemical measures in biopsied skin and blubber tissues. Biopsies are collected annually from distinct southern hemisphere populations, whilst they are in their tropical breeding grounds. The first 10 years of monitoring of the E1 population under the HWSP offers a unique opportunity to investigate dietary heterogeneity within healthy, free-roaming adult individuals of the same breeding population and enhance our understanding of lipids and fatty acids in the adipose tissue of E1 humpback whales. The 10-year timeline allows for the assessment of interannual variability and potential directional changes in the diet of E1 humpback whales, which are sampled during their northward migration to the breeding grounds in June and July, and during their southward migration to the feeding grounds in September and October. As such, the specific aims of this study were to: (1) determine if there is interannual variability in the lipid and fatty acid profiles of E1 humpback whales, (2) whether fatty acid profiles can be used to distinguish sampling cohorts, and (3) if interannual variability can be explained by environmental changes in the corresponding Southern Ocean feeding ground of the E1 population.

Based on the classical feeding paradigm, it can be hypothesised that there should be only a small degree of interannual variability in the fatty acid profiles of E1 humpback whales abiding by a high-fidelity Antarctic krill diet. This investigation thereby serves as a guide to enhance our understanding of the potential variability in the feeding ecology of E1 humpback whales, and to further enhance interpretation of dietary tracer signals.

## Results

To explore population heterogeneity and temporal variation in the diet of E1 humpback whales, outer blubber lipid components were measured in blubber biopsies collected from outwardly healthy, free-roaming, adult individuals between 2008 and 2018.

### Lipid content

The average outer blubber total lipid content appeared to fluctuate among sampling years with a minimum average total lipid content observed in 2009 (35.5 ± 14.3%, n = 26) and 2014 (35.5 ± 16.6%, n = 38), and a maximum average total lipid content in 2016 (56.7 ± 14.8%, n = 63; Fig. S1). The difference among years was significant (PERMANOVA: pseudo-*F*_8, 346_ = 8.5542; *p* = 0.0001, n = 348) and 47.34% of the total variation in average total lipid content was explained by factor “year”. A *post-hoc* pairwise analysis showed that half of the years differed significantly from each other (PERMANOVA: *p* < 0.05), while the other half did not. Each year differed significantly from 2016, while 2009 and 2014 also differed significantly from 2008, 2013, 2017 and 2018 (Table S1). In those years, when both migration time-points were captured (2008, 2009, 2016, 2017, 2018), the average total lipid content of the northward migration was higher than the average total lipid content of the southward migration, with the exception of 2018 where the average total lipid content of southward migrating whales was higher than that of northward migrating whales (Fig. S1). A PERMANOVA with year and migration as fixed factors showed that the interaction between year and migration was significant (PERMANOVA: pseudo-*F*_4, 341_ = 2.6832; *p* < 0.0295, n = 194). A *post-hoc* analysis revealed a significant difference among all other years and 2016 for the northward migration, but not for the southward migration (PERMANOVA: *p* < 0.05; Table S1).

### Fatty acid classes

The fatty acid profiles of E1 humpback whales were dominated by monounsaturated fatty acids (MUFA), with shorter chain MUFA (≤ C_18_ carbon atoms, SC-MUFA) making up a bigger proportion than long-chain MUFA (> C_20_ carbon atoms, LC-MUFA, Table 1). The difference among fatty acid classes (saturated fatty acids, SFA; MUFA; polyunsaturated fatty acids, PUFA) was significant (χ^2^_4_ = 1437.7, *p* < 0.05, n = 348) and a post-hoc analysis revealed that all fatty acid classes were significantly different from each other. There appeared to be no obvious pattern among years or between migrations, and neither factor showed a significant effect (Kruskal–Wallis: Year: χ^2^_8_ = 1.74, *p* = 0.9881, n = 348; Migration: χ^2^_1_ = 0.17, *p* = 0.6794, n = 194).

**Table 1:** Relative abundance of fatty acids (FA) as percent of total FA (means and standard error of the mean) in E1 humpback whales from 2008 to 2018 (n = 348). Fatty acids listed under “Other FA” are present in less than trace amounts (≥ 0.5%). SFA: saturated FA; MUFA: monounsaturated FA; PUFA: polyunsaturated FA; SC: shorter chain; LC: long-chain; N: number of whales sampled during north migration; S: number of whales sampled during south migration.

Fatty Acids (FAs)	2008 (N = 13) (S = 9)	2009 (N = 11) (S=14)	2011 (N = 0) (S =26)	2013 (N=0) (S=31)	2014 (N = 33) (S = 0)	2015 (N = 0) (S = 64)	2016 (N = 49) (S=14)	2017 (N = 33) (S = 16)	2018 (N = 12) (S =23)
**Saturated **									
14:0	8.55 ± 1.22	6.21 ± 0.34	6.94 ± 0.38	7.13 ± 0.34	8.01 ± 0.33	8.45 ± 0.14	9.81 ± 0.27	8.34 ± 0.18	8.55 ± 0.2
16:0	15.44 ± 1.4	11.37 ± 0.41	11.63 ± 0.41	11.29 ± 0 43	12.99 ± 0.44	11.57 ± 0.18	15.33 ± 0.54	12.54 ± 0.31	12.7 ± 0.24
18:0	3.25 ± 0.54	2.32 ± 0.15	2.09 ± 0 1	1.98 ± 0 1	2.91 ± 0.44	1.86 ± 0.04	2.66 ± 0.13	2.03 ± 0.06	2.01 ± 0.05
**% Total SFA **	**22.18 ± 3.47**	**24.76 ± 6.08**	**21.99 ± 3.62**	**21.48 ± 4.45**	**25.73 ± 4.36**	**22.9 ± 2.68**	**29.76 ± 7.79**	**24.27 ± 3.78**	**26.0 ± 2.82**
**Monounsaturated**									
14:1	0	0	0	1.74 ± 0.07	1.42 ± 0.09	1.81 ± 0.05	1.41 ± 0.06	1.49 ± 0.08	0.65 ± 0.05
16:1ω7c	22.06 ± 2.51	12.41 ± 0.69	16 02 ± 0.87	17.55 ± 0.53	15.71 ± 0.47	15.15 ± 0.27	14.19 ± 05	14.92 ± 0.43	14.16 ± 0.46
18:1ω9c	32.82 ± 2.89	22 ± 0.49	26.67 ± 1.11	25.9 ± 0.57	24.89 ± 0.42	25 ± 0.22	23.63 ± 0.66	21 22 ± 0 59	24.87 ± 0.37
18:1ω7c	12.27 ± 1.13	6.4 ± 0.15	9.8 ± 0.5	8 63 ± 0.33	8.75 ± 0.11	8.36 ± 0.09	9.58 ± 0.43	9.04 ± 0.41	8.46 ± 0.18
20:1ω9	2.51 ± 0.51	2.01 ± 0.17	1.93 ± 0.11	1.49 ± 0.07	1.36 ± 0.08	1.63 ± 0.07	1.67 ± 0.07	1.37 ± 0.05	1.86 ± 0.09
**% Total SC MUFA **	**55.26 ± 6.43**	**45.59 ± 5.73**	**57.47 ± 4.78**	**56.64 ± 5.64**	**53.35 ± 5.01**	**53.15 ± 3.55**	**53.27 ± 4.47**	**49.81 ± 4.18**	**51.65 ± 3.22**
**% Total LC MUFA **	**3.25 ± 1.84**	**4.48 ± 2.19**	**2.82 ± 0.66**	**2.28 ± 0.57**	**2.31 ± 0.63**	**2.79 ± 0.71**	**2.26 ± 1.52**	**1.85 ± 0.43**	**2.05 ± 0.59**
**Polyunsaturated**									
18:4ω3	1 ± 0.16	0.8 ± 0.06	0.69 ± 0.05	0.71 ± 0.04	0.68 ± 0.06	0.87 ± 0.03	0.68 ± 0.06	1.05 ± 0.05	0.97 ± 0.03
18:3ω3	0.72 ± 0.08	0.45 ± 0.03	0.51 ± 0.03	0.54 ± 0.02	0.65 ± 0.05	0.53 ± 0.01	0.4 ± 0.03	0 58 ± 0.02	0.55 ± 0.02
18:2ω6	3.66 ± 0.5	2.39 ± 0.05	2.57 ± 0.11	2.54 ± 0.05	2.32 ± 0.08	2.55 ± 0.01	1.97 ± 0.11	2.56 ± 0.05	2.54 ± 0.03
20:4ω6	0.66 ± 0.1	0.54 ± 0.03	0.42 ± 0.02	0.52 ± 0.02	0.5 ± 0.07	0.49 ± 0.01	0.32 ± 0.04	0.51 ± 0.02	1.47 ± 0.38
20:5ω3	8.36 ± 1.29	7.28 ± 0.44	5.25 ± 0.33	5.65 ± 0.24	5.18 ± 0.36	6.16 ± 0.11	4.28 ± 0.36	7.37 ± 0.23	5.66 ± 0.45
20:4ω3	1.16 ± 0.22	1 12 ± 0.05	0.87 ± 0.05	0.97 ± 0.05	0.89 ± 0.06	0.93 ± 0.02	0.68 ± 0.04	1.09 ± 0.04	0.96 ± 0.05
21:5ω3	0.34 ± 0.05	0.28 ± 0.02	0	0.2 ± 0.01	0	0	0.18 ± 0.02	4.12 ± 0.11	0
22:6ω3	5.57 ± 0.84	5.53 ± 0.24	3.55 ± 0.22	3.94 ± 0.2	3.57 ± 0.28	4 86 ± 0.11	3.24 ± 0.29	5 65 ± 0.19	5.34 ± 0.18
22:5ω3	4.98 ± 0.77	4.53 ± 0.24	3.27 ± 0.19	3.52 ± 0.16	3.31 ± 0.24	3.63 ± 0.1	2.41 ± 0.21	4.11 ± 0.13	3.73 ± 0.15
**% Total SC PUFA **	**3.91 ± 1.46**	**3.76 ± 0.63**	**3.90 ± 0.87**	**4.01 ± 0.61**	**4.38 ± 1.13**	**4.13 ± 0.29**	**3.21 ± 1.52**	**4.40 ± 0.70**	**1.65 ± 0.26**
**% Total LC PUFA **	**15.41 ± 6.34**	**21.4 ± 14.05**	**13.8 ± 13.78**	**15.59 ± 3.49**	**14.23 ± 5.39**	**17.03 ± 2.21**	**11.51 ± 7.57**	**19.66 ± 3.93**	**18.66 ± 2.52**
**% Total Omega 3**	**15.48 ± 6.39**	**20.32 ± 4.5**	**14.62 ± 4.02**	**15.96 ± 3.6**	**14.74 ± 5.59**	**17.96 ± 2.31**	**12.29 ± 8.0**	**20.85 ± 4.19**	**18.7 ± 14.03**
**% Total Omega 6**	**3.71 ± 1.58**	**4.17 ± 0.63**	**3.09 ± 0.67**	**3.51 ± 0.41**	**3.25 ± 0.94**	**3.17 ± 0.13**	**2.42 ± 1.13**	**3.22 ± 0.5**	**1.59 ± 2.46**
Other FA^1^									
**FA ratios**									
16:1ω7c/16:0	1.41 ± 0.09	1.19 ± 0.12	1.45 ± 0.11	1.68 ± 0.13	12.6 ± 0.07	1.35 ± 0.05	1 .00 ± 0.05	1.24 ± 0.06	1.15 ± 0.06
18:1ω7c/18:1ω9c	0.36 ± 0.01	0.38 ± 0.01	0.55 ± 0.2	0.34 ± 0.005	0.35 ± 0.004	0.34 ± 0.02	0.36 ± 0.02	0.55 ± 0.12	0.56 ± 0.03
20:5ω3/22:6ω3	1.51 ± 0.06	1.31 ± 0.05	1.46 ± 0.05	1.51 ± 0.06	1.77 ± 0.23	1.29 ± 0.03	16 ± 0.07	1.32 ± 0.02	1.07 ± 0.09
**Carnivory Index **									
Carn./∑ herb. ± carn.	0.49 ± 0.01	0.51 ± 0.01	0.49 ± 0.02	0.49 ± 0.01	0.5 ± 0.005	051 ± 0.004	0.49 ± 0.01	0.46 ± 0.01	051 ± 0.01

Bold values indicate the total percentage of FAs in each FA class.

^1^ i14:0, i15:0, 15:0, i17:0, 17:0, i18:0, 20:0, 22:0,14:1ω5c, 16:1ω9c, 16:1ω7t, 16:1ω5c, 18:1ω7t, 18:1ω5c, 18:1, 19:1b,20:1ω7c, 20:1ω7c, 22:1ω11c, 22:1ω9c, 22:1ω7c, 24:1ω9c, 16:3, 20:3ω6, 20:2ω6, 22:4ω6,24:6ω3 .

### Non-dietary fatty acids

In all sampled E1 humpback whales, 50 different fatty acids were identified. Of these, 17 were consistently found in greater than trace amounts (≥ 0.5%) and accounted for 96–97% of total fatty acids. The top five most abundant fatty acids were all SFAs and MUFAs and were consistent each year, except 2009 (Table 1). 18:1ω9c, 16:1ω7c, 16:0, 18:1ω7c and 14:0 were the dominant fatty acids in decreasing order of relative abundance (Table 1). In 2009, EPA, a dietary fatty acid, was more abundant than 14:0 (Table 1). The top five fatty acids made up around 65–70% in north and south migrating whales in all years, except 2008 (Table 1). In 2008, they made up almost 100% in north migrating humpback whales and around 80% in south migrating whales (Table 1).

### Dietary fatty acids

**Figure 1:**
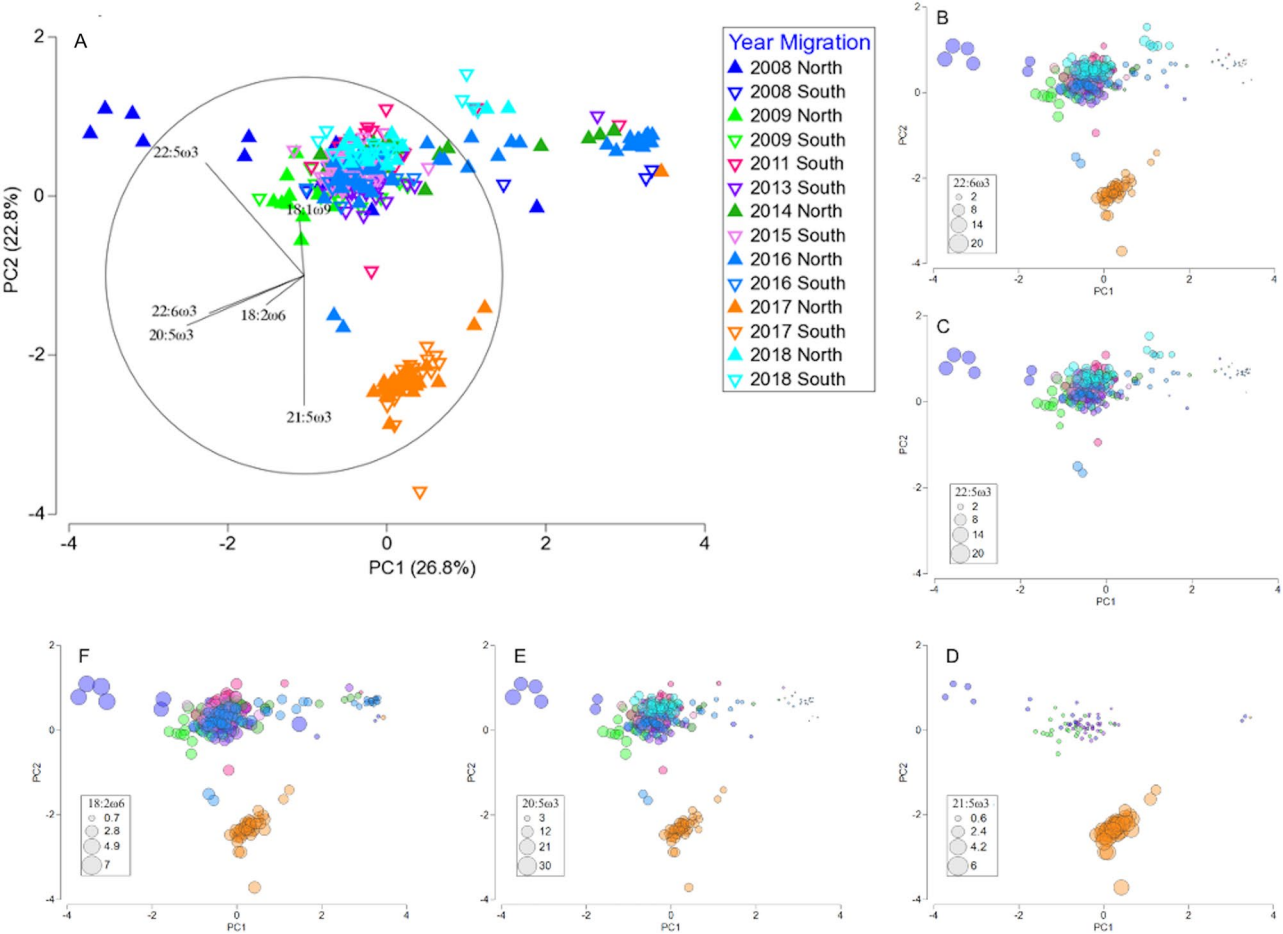
Principal component analysis (PCA) of fatty acid composition of E1 humpback whales from 2008 to 2018 (**A**). Graphs (**B**–**F**) show the same PCA with a bubble plot overlayed, showing the percentage of individual, dietary fatty acids (n = 348).

Principal component analysis was performed on fatty acid percentages to investigate the major drivers of variability among years. Only those fatty acids that accounted for more than 0.5% of the total fatty acid profile were included in the analysis. The first two principal components (PC1 and PC2) explained 49.6% of the variation among years (Fig. 1A). All years, except 2017 were spread out in a line along PC1, with PC2 clearly separating 2017 from the other years (Fig. 1A). This was mainly attributed to the very low percentage of docosapentaenoic acid (DPA; 22:5ω3) and the presence of 21:5ω3 (Fig. 1C,D; Table 1). The percentage of EPA in southward migrating humpback whales was also greater in 2017 than any other year (Table 1). The fatty acid, 21:5ω3 had the highest loading on PC2, clearly indicating that this fatty acid was the major driver separating 2017 from the other years (Fig. 1, Table S2). Eicosapentaenoic acid had the highest loading on PC1, contributing to the spread of all years along PC1, except 2017 (Fig. 1, Table S2). The spread along PC1 is further driven by samples on the left-hand side of the PCA having consistently higher percentages of dietary fatty acids than those on the right-hand side of the PCA (Fig. 1B–F). Only one of the top five dominant fatty acids, 18:1ω9c was a driver in separating the years from each other in the PCA (Fig. 1). All other major drivers in the separation of years in the PCA were dietary fatty acids, including the two Antarctic krill indicator fatty acids, EPA and DHA. A PERMANOVA with year and migration as fixed factors and total lipid content as a covariate showed that the interaction of year and migration was significant (PERMANOVA: pseudo-*F*_4, 330_ = 7.2285, *p* = 0.001) as was the covariate total lipid content (PERMANOVA: pseudo-*F*_1, 330_ = 15.431, *p* = 0.001). A pairwise comparison showed that all years differed significantly from each other (PERMANOVA, *p* < 0.05), and that the north and south migration only differed significantly from each other in 2008, 2009 and 2016 (Table S3).

**Figure 2:**
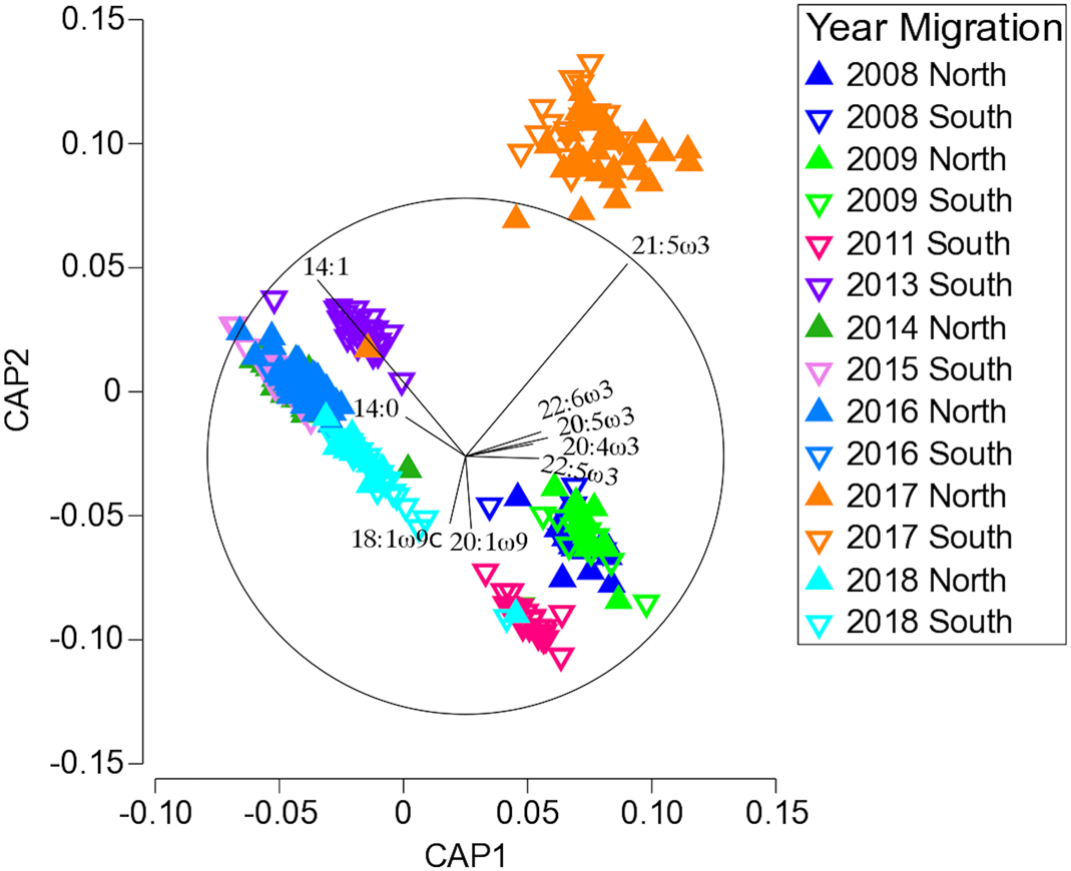
Canonical analysis of principal coordinates (CAP) showing the fatty acid percentage composition of north (closed upward arrow) and south (open downward arrow) migrating E1 humpback whales from 2008 to 2018 (n = 348).

As the PCA did not sufficiently separate years, but the PERMANOVA indicated that there was a significant difference among years and between migrations, a canonical analysis of principal coordinates (CAP) was performed. This analysis is used for finding an axis through the multivariate data cloud that best separates the groups rather than tests for differences among them. The CAP analysis showed that the years can be separated by fatty acid profiles (Fig. 2). The CAP analysis clearly separated 2011, 2013, 2017 and 2018 from the other years. It also showed that 2008 and 2009 clustered together while 2014, 2015 and 2016 formed a separate cluster (Fig. 2). Overall, 78.84% of samples (n = 279/345) were correctly classified by the CAP cross validation procedure into the year that they were sampled in (Table S4. The lowest classification success was recorded for the years 2008, 2014 and 2016, with only 54–58% of samples being correctly classified to the respective year. The classification success for all other years ranged from 74 – 98%, with 2017 having a mis-classification error of 2% (Table S4).

Both the PCA of fatty acid profiles and subsequent CAP analysis revealed that the PUFA of dietary origin are the driving factors in separating the sampling years from each other. Three MUFA, 14:1, 18:1ω9c and 20:1ω9, played a role in separating the years from each other, but all with minor loadings (Table S2). Outliers of higher or lower percentages than the 10-year average mainly occur among the dietary fatty acids rather than the SFA or MUFA. In 2008, 2009 and 2017, whales had higher than average percentages of EPA and DPA, while in 2016 whales had lower than average percentages of EPA and DPA (Table 1). Fatty acid profiles from 2017 whales showed a much higher than average percentage of 21:5ω3. In 2008 whales, 18:2ω6 was present at a higher than average percentage, while it was present at a lower than average percentage in the 2016 whales. In the 2018 whales, the percentage of 20:4ω6 was three times higher than the average percentage of all other years (Table 1). All aforementioned fatty acids are drivers in the separation of sampling cohorts, as fatty acid profiles of all sampling years differ significantly from each other. Indeed, both the PCA and CAP analysis show that 21:5ω3 was the major driver, separating 2017 whales very clearly from all other years, resulting in a classification success of 98% for 2017 in the CAP cross validation procedure (Table S4).

### Diet investigation

**Figure 3:**
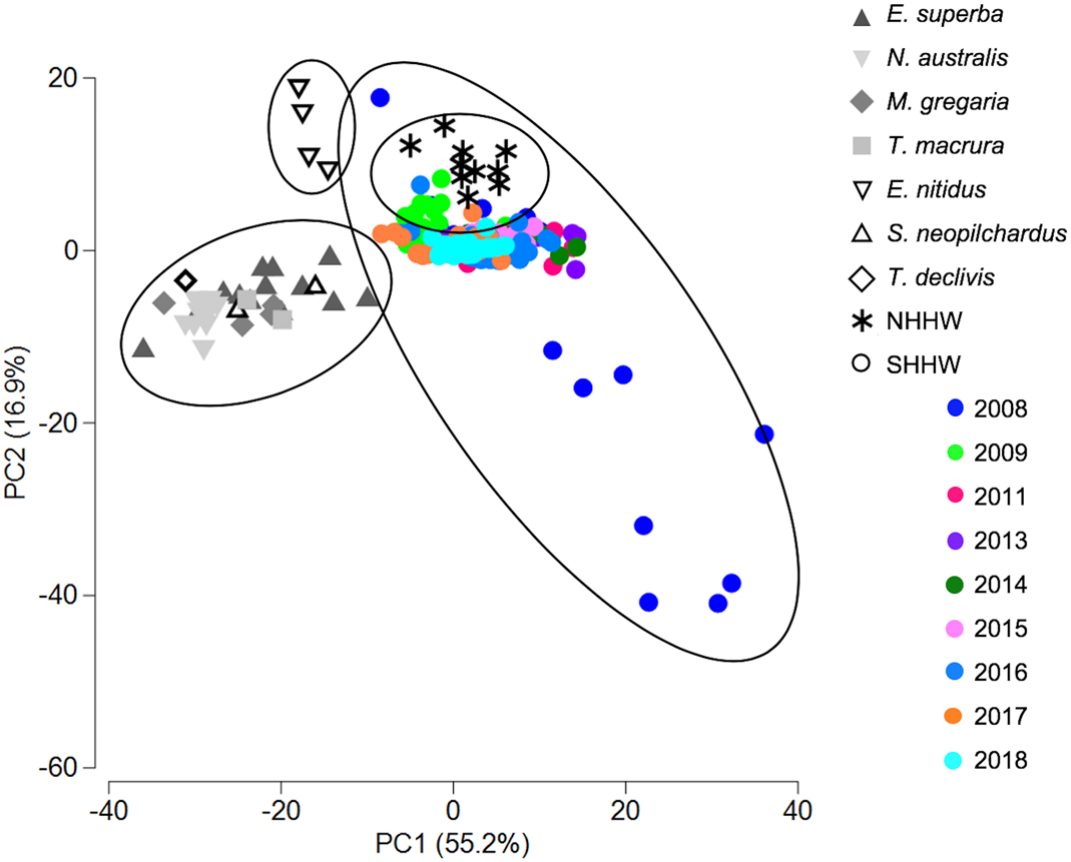
Principal Component Analysis (PCA) of fatty acid composition of E1 humpback whales from 2008 to 2018 (this study), northern hemisphere humpback whales (data from Borobia et al.^29^), Antarctic krill (data from Phleger et al.^23^; Stübing and Hagen^22^; Guang et al.^17^), temperate krill (data from Virtue et al.^26^) and other prey species (*T. macrura*: Guang et al.^17^; Mayzaud et al.^19^; Kattner et al.^77^; *M. gregaria*: Phillips et al.^25^; Varisco et al.^24^; *E. nitidus*, *S. neopilchardus*, *T. declivis*: Nichols et al.^28^; Baylis et al.^27^).

**Figure 4:**
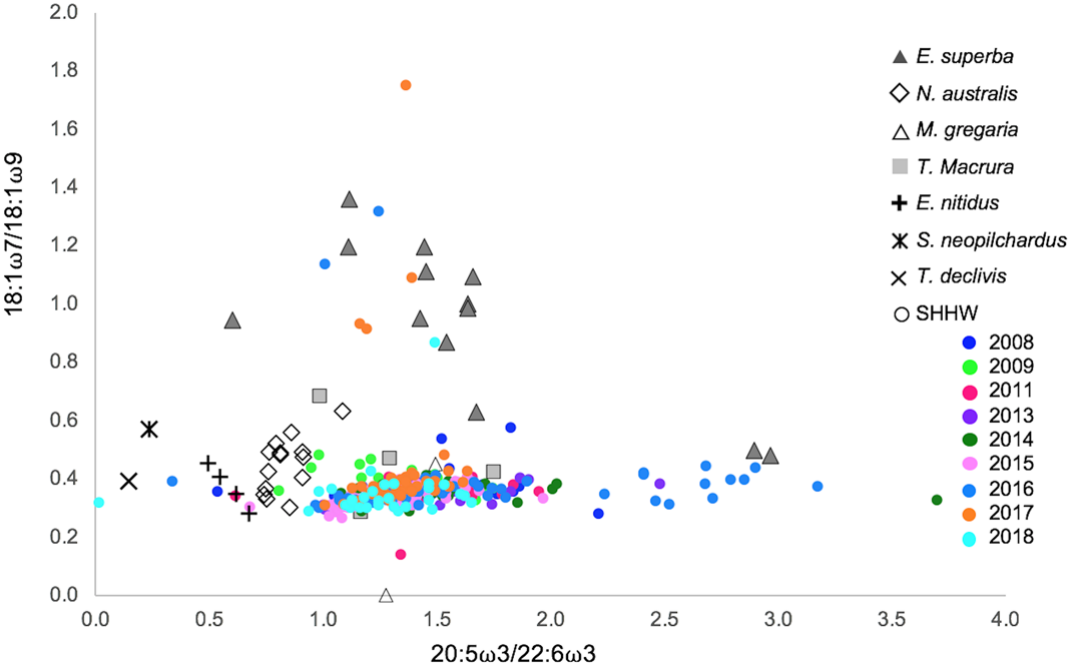
Scatterplot of fatty acid ratios of vaccenic acid to oleic acid (18:1ω7c/18:1ω9c) and EPA to DHA of E1 humpback whales from 2008 to 2018 (this study), northern hemisphere humpback whales (data from Borobia et al.^29^), Antarctic krill (data from Phleger et al.^23^; Stübing and Hagen^22^; Guang et al.^17^), temperate krill (data from Virtue et al.^26^) and other prey species (*T. macrura*: Guang et al.^17^; Mayzaud et al.^19^; Kattner et al.^77^; *M. gregaria*: Phillips et al.^25^; Varisco et al.^24^; *E. nitidus*, *S. neopilchardus*, *T. declivis*: Nichols et al.^28^; Baylis et al.^27^).

**Figure 5:**
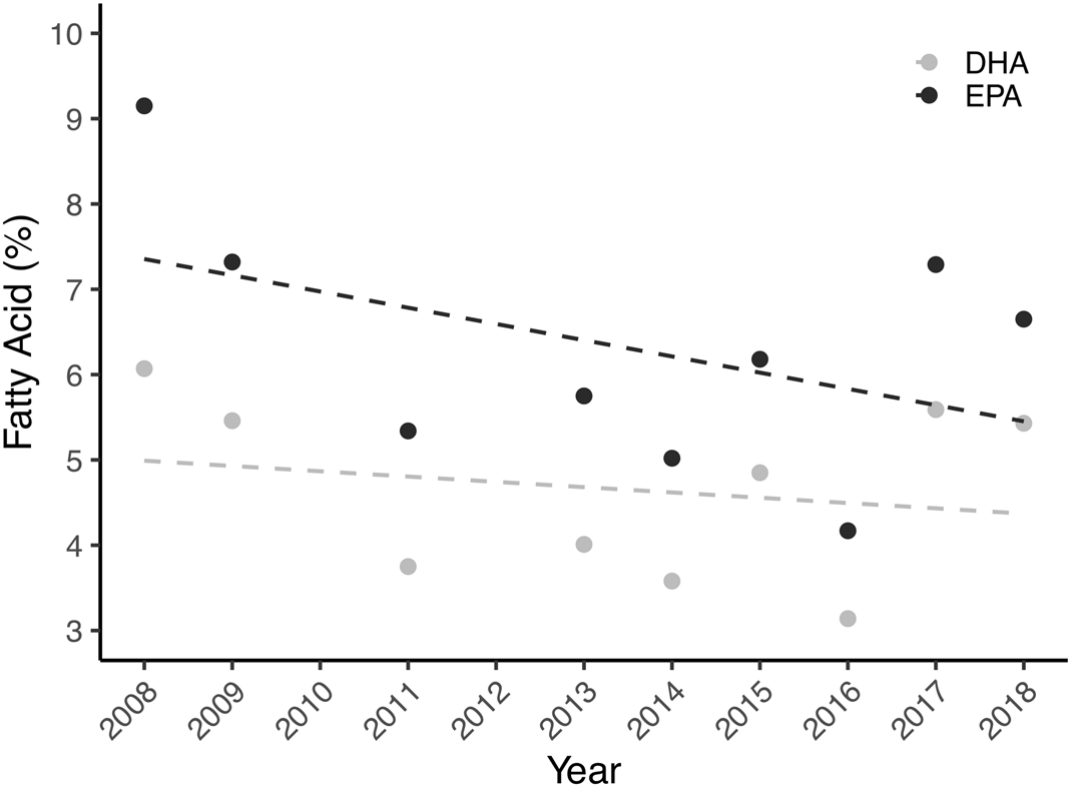
Scatterplot of the averages of eicosapentaenoic acid (20:5ω3) and docosahexaenoic acid (22:6ω3) of E1 humpback whales from 2008 to 2018 (n = 348).

Relationships between E1 humpback whales, their potential prey (data sources: *Thysanoessa macrura*:^17–19^; *E. superba*:^20–23^; *Munida gregaria*:^24,25^; *N. australis*:^26^; *Emmelichthys nitidus*, *Sardinops neopilchardus*, *Trachurus declivis*:^27,28^) and northern hemisphere humpback whales (data source:^29^) were evaluated by PCA using the 10 major fatty acids reported in all studies. The majority of fatty acid profiles of E1 humpback whales formed a distinct cluster, with some samples taken in 2008 representing outliers (Fig. 3). The PCA displayed a clear distinction between the main cluster of E1 humpback whales, their potential prey species and northern hemisphere humpback whales (Fig. 3). Together, PC1 and PC2 accounted for 72.1% of the variance among fatty acid profiles of the three groups (Fig. 3). A PERMANOVA confirmed that the observed differences were statistically significant (pseudo-*F*_1, 330_ = 15.431, *p* = 0.001). A scatterplot of the fatty acid ratios of vaccenic acid to oleic acid (18:1ω7c/18:1ω9c) and EPA to DHA was used to investigate the trophic level of E1 humpback whales. The results showed that Antarctic krill have a higher 18:1ω7c/18:1ω9c ratio than E1 humpback whales or any other of their potential prey species (Fig. 4). Additionally, the EPA to DHA ratio of E1 humpback whales and Antarctic krill was higher than that of *N. australis* (Fig. 4). A second scatterplot of the averages of EPA and DHA from 2008 to 2018 showed that the proportion of EPA in fatty acid profiles of E1 humpback whales declined slightly (*t*_7_ = − 0.385, *p* = 0.356), but not significantly while the proportion of DHA remained constant (*t*_7_ = − 0.177, *p* = 0.432; Fig. 5). The ratio of 16:1ω7c/16:0 was above 1 in all sampling years and the CI index ranged from 0.46 and 0.51 (Table 1).

### Environmental model

The DistLM results showed that the model that included all five environmental variables (CHL, ENSO, SAM, Sea ice concentration, SST) had the highest adjusted *R*^2^ value (0.25) and hence provided the best model solution. The model that only included the dietary fatty acids as the dependent variable had a higher adjusted *R*^*2*^ value (0.32) than the model that included all fatty acids as the dependent variable (0.25). The models that included either a 1-year lag phase or 2-year lag phase had lower adjusted *R*^2^ values than the within-year model. The model, only including dietary fatty acids without a lag phase explained 33% of the total variation in fatty acid profiles of E1 humpback whales from 2008 to 2018. In this model, 23.7% of the variability was explained by SAM, while 5% of the variation was explained by SST. The remaining three factors explained less than 3% each.

## Discussion

This study presents the first decadal examination of variation in lipid and fatty acid profiles in any humpback whale population using the same methods throughout the entire study period. Such long-term records enable inferences about interannual and cyclical variation in diet and demonstrate the utility of fatty acid profiles as a metric to distinguish among sampling cohorts. Both total lipid content and fatty acid profiles show temporal variation in the diet of E1 humpback whales. While fatty acid profiles showed a significant difference among all years, total lipid content only showed a significant difference among half of the sampling years, indicating that fatty acid profiles provide a more reliable metric to distinguish among sampling cohorts than lipids. Finding such prominent interannual variability with distinct year to year differences in the fatty acid profiles of a high-fidelity Antarctic krill consumer was not expected, and thus poses the question whether this variation originates from a more diverse diet of E1 humpback whales, or changes in the diet of their main prey item, Antarctic krill.

### Lipid content

Our results indicate that interannual variability in total lipid content is present in E1 humpback whales. The years that vary significantly from each other, are those with a very high or very low total lipid content, compared to the average. With the exception of 2018, our results align with previous results that showed an average decrease of 23% between northward and southward sampling windows for this population^30^. This corresponds with the estimated 30–50% reduction in post-summer feeding reserves of southern hemisphere humpback whales across the migration season^31^. The discrepancy observed in 2018 could be a sampling artefact because the sample size in the north migration was relatively small (n = 12). The sample set included three whales that had a total lipid content well below average for northward migrating whales. In addition, modelling indicated that when samples from each year were separated into north and south migrating sampling cohorts, only 17.17% of the interannual variability of total lipid content was explained by the factors “year” and “migration” combined. The remaining, unexplained variability may be an artefact of the fasting life history strategy of humpback whales, sampling method and sample size. Percent blubber lipid content has often been used as a proxy for body condition in large, free-roaming cetaceans^32^, however, the limitations of this approach, particularly when measuring total lipid content from small amounts of remotely biopsied outer blubber tissue, have recently received attention in the literature^32–35^. The results of this study further emphasise the loose, but imperfect, relationship between body condition and outer blubber lipid content measured via remote biopsy highlighted in other studies.

### Fatty acid classes

The proportions of fatty acids remain constant, independent of total lipid content in a biopsy sample^36^ and were found here to provide a more reliable metric to distinguish among sampling cohorts than total lipid content. When just looking at fatty acid classes, there was a significant difference among classes but there was no obvious pattern among years or between migrations. Shorter chain MUFA made up the largest proportion in each sampling year (Table 1). They are commonly reported as the major fatty acid class in the outer blubber layer of cetaceans^37,38^, as they play an important role in the thermoregulatory function of this blubber layer. Due to the fact that the melting point of fatty acids decreases with decreasing carbon chain length, tissue fluidity of the outer blubber layer is enhanced with a larger proportion of shorter chain MUFA^39^. As thermoregulation is one of the biggest challenges for aquatic mammals, maintaining fluidity and thickness of the outer blubber layer is crucial to avoid increased energetic costs associated with maintaining thermal balance^40^. Our results showing no difference in fatty acid classes among years or between migrations support the maintenance role of the outer blubber layer.

### Non-dietary fatty acids

The importance of the thermoregulatory role of the outer blubber layer is highlighted even further by the fact that the four most dominant fatty acids found in this study are the same as in a number of other marine mammal species. Several Mysticeti, Odontoceti and Pinniped species have 18:1ω9c, 16:1ω7c, 16:0 and 18:1ω7c as the dominant fatty acids in the outer blubber layer^41–45^. All four fatty acids are either SFA or MUFA, which are more stable and less susceptible to oxidation than PUFA. Additionally, all four are endogenous and can be readily synthesised by the animal if necessary^46^. However, de novo synthesis of fatty acids in mammals usually only occurs when the animal is consuming a low-fat diet^15^, which is not the case for humpback whales, as Antarctic krill are rich in lipids^47^, particularly in 16:1ω7c as they have a high dietary uptake and low excretion rate of this fatty acid^48^. Thus, it is unlikely that de novo synthesis plays a role in the dominance of shorter chain MUFA in the outer blubber layer, especially in humpback whales, as fasting animals do not synthesis or modify their fat stores, but rather mobilise and completely oxidise them for energy^15^. As de novo synthesis, elongation or desaturation of fatty acids is limited to SFA and MUFA, and generally inhibited during fasting^15^, it is likely that the fatty acid profiles studied here, are representative of the time of deposition and hence the diet that the humpback whales consumed in Antarctica.

### Diet investigation

The whales’ ratio of 18:1ω7c/18:1ω9c is similar to that of all potential prey species, except Antarctic krill. These results are consistent with a consumer-prey relationship occurring between E1 humpback whales and Antarctic krill. This ratio is generally used to distinguish between a herbivorous and carnivorous diet^49,50^. Our results show that E1 humpback whales, their potential temperate prey species (*N. australis*, *E. nitidus*, *S. neopilchardus*, *T. declivis*) and their potential Antarctic prey species (*T. macrura* and *M. gregaria*) all occupy a more carnivorous feeding niche than Antarctic krill, as they have lower 18:1ω7c/18:1ω9c ratios. The CI of E1 humpback whales ranges between 0.46 and 0.51, which is indicative of an omnivorous diet. These results show that E1 humpback whales feed at a relatively low trophic level and provide evidence against substantial energy acquisition via alternate prey as this would lower the whale’s 18:1ω7c/18:1ω9c ratio and increase their CI.

The ratio of EPA/DHA can also be used to determine the degree of carnivory as DHA is conserved throughout the food web and as a result, the ratio of EPA/DHA should be lower at higher trophic levels^51,52^. Here, E1 humpback whales have higher EPA/DHA ratios than any of the temperate prey species, lending support that E1 humpback whales feed at a similar or even lower trophic level than some zooplankton species. Usually, the ratio of EPA/DHA is used to distinguish between a diatom and a dinoflagellate-based diet, with a high ratio reflecting a diatom diet and a low ratio reflecting a dinoflagellate diet^22,23,48^. Hence, the high ratio of EPA/DHA observed here in E1 humpback whales and Antarctic krill indicates that they both derive energy from a diatom-origin diet, whereas the lower ratios observed in all temperate species indicate that they derive energy from a dinoflagellate-origin diet. Values above 1 of the ratio 16:1ω7c/16:0 in E1 humpback whales also imply that a significant part of the diet has a diatom-origin source^52^. In addition, high levels of 16:1ω7 and low levels of 18:4ω3, as present in E1 humpback whales, are also an indication of a diatom-origin diet^53^. These results lend support to the view that E1 humpback whales show high-fidelity to an Antarctic krill diet. This assumption is reinforced by the relatively constant proportion of the two indicator fatty acids for Antarctic krill, EPA and DHA, from 2008 to 2018. In fact, there was no change in the average proportion of DHA, and only a small, non-significant decrease in the average proportion of EPA. Combined, these findings support the classical feeding paradigm that principal energy acquisition for E1 humpback whales occurs through the consumption of Antarctic krill.

However, feeding on temperate prey species may be masked in the fatty acid profiles presented in this study because whales were sampled arriving from their Antarctic feeding grounds, close to their breeding grounds. Temperate feeding in E1 humpback whales has been observed further south along the migration route and only in southward migrating whales. Therefore, it remains unclear if indications of feeding in temperate waters in the fatty acid profiles of E1 humpback whales would be present in whales sampled in late spring upon their return to the Southern Ocean. Indications of feeding in temperate waters the year prior to sampling may be masked by intense summer feeding on Antarctic krill.

Humpback whales are secondary consumers, therefore interannual variability in the fatty acid profiles can either be influenced by changes in the whales’ diet, or by changes in the diet of their prey. Our results show that the prominent interannual variability is mainly driven by dietary fatty acids, which are generally thought to be deposited unmodified into the adipose tissue of marine mammals, in similar proportions to that consumed in the diet^15^. As such, interannual variability points to changes in the diet of Antarctic krill. Our results do, however, show that there is a distinct difference between the fatty acid profiles of E1 humpback whales and their potential prey species, which indicates that species-specific metabolism has an influence on the fatty acid profiles of E1 humpback whales and that fatty acids are not deposited unmodified after ingestion. Our result corresponds to prior research on other marine mammal species that found that the adipose tissue fatty acid composition never exactly matches that of the diet^54^. Nevertheless, diet does have an impact on the fatty acid profiles of humpback whales, as our results show a significant difference between E1 humpback whales and northern hemisphere humpback whales, which have a much broader diet and feed at higher trophic levels and often at lower latitudes^55^.

Antarctic krill experience a diverse range of environmental conditions due to seasonal, annual and geographical change. Their diet also varies on a seasonal, annual^23,56,57^, and regional basis^58,59^. Seasonal and interannual variability in the diet of Antarctic krill can be observed in their fatty acid profiles^23,56,57^. Geographical differences have been mainly observed in gut content analyses^58,59^, but only weakly in fatty acid profiles, despite the phytoplankton community varying markedly across the same geographical range^48,60^.

Seasonal variability is most evident in the fatty acid profiles of Antarctic krill, with interannual variability seen in proportional changes of specific fatty acids as well. Interannual variability is particularly pronounced in 18:4ω3, as it has been shown to vary between 0–11% in the fatty acid profile of Antarctic krill in multiple studies^21,61^. This trend indicates that 18:4ω3, a dinoflagellate biomarker in the diet of Antarctic krill, could play a large role in determining the overall fatty acid profile of the humpback whale’s main prey species^56^. Interestingly, this fatty acid was present in high percentages in Antarctic krill sampled around the Western Antarctic Peninsula, South Georgia and the South Orkney Islands in the summer of 2016/2017 (Fig. 1)^56^, and in E1 humpback whales in the 2017 sampling year (Table 1). As sampling of Antarctic krill is rarely conducted in the east Antarctic feeding area attributed to the E1 humpback whale breeding population (Fig. 1)^10^, it is unknown if the trend of high 18:4ω3 percentages was localised to the Western Antarctic Peninsula (WAP) or similarly present in Antarctic krill throughout the Southern Ocean during the 2016/2017 austral summer. This precludes direct linking of high percentages of major dietary fatty acids in E1 humpback whales and those observed in Antarctic krill from the WAP. Nonetheless, it raises such a possibility. The presence of interannual variability in the fatty acid profiles of both, E1 humpback whales and their main prey, further suggest an underlying environmental driver of such variability.

### Environmental model

Here, only 33% of the interannual variability in fatty acid profiles of E1 humpback whales was explained by two environmental indices (SAM, ENSO) and three abiotic measures (sea ice concentration, SST, CHL) included in a discriminant analysis distance based linear model. The model that only included dietary fatty acids and no lag phase had the best model fit, suggesting again that there is more variability among dietary fatty acids than non-dietary fatty acids. Overall, the model provides a low explanatory power, but indicates that large scale, periodic, cyclical climate variations in wind and sea surface temperature, and in turn upwelling regimes have the highest influence on fatty acid profiles. The low explanatory power of this model is surprising considering that there was a direct covariance observed between ENSO and abiotic measures (sea ice concentration and CHL), and the HWSP adiposity markers, namely the outer blubber persistent organic pollutant concentration and the Adipocyte Index (AI) across a sub-set of this time-line for the same population^9^. An alternate dietary tracer under the HWSP,^13^C and ^15^N bulk stable isotopes, in the same study revealed signs of foraging diversification following lean years^9^, consistent with a lag phase in response. Notably, however, fatty acid profiles of Antarctic krill have also been shown to correlate with ENSO and sea ice extent around the South Shetland islands^57^ and neither a lag phase, nor exclusion of SST improved the explanatory power of our model as the best model fit with the highest explanatory power in this study included all five aforementioned abiotic variables. These results emphasise the different influences upon different environmental tracers and the strength of using a suite of tracers in combination.

In line with evidence of fatty acid profile variability being attributed to lower levels of the food web, model findings underscore the complexity of fatty acids and their application for dietary studies. Complexity in using fatty acids as a biochemical tracer of diet arises from the great diversity of the commonly 50 to 70 different fatty acids being present in marine mammals, as well as the different metabolic pathways in which fatty acids can be synthesised, desaturated or elongated prior to, or post deposition^15^. In addition to this, the explanatory power of our model was reduced by the complexity of different long-term and interannual environmental conditions in the Southern Ocean and their impact on the phytoplankton community and hence the primary and secondary consumers^9,57^. Incorporating two complex systems into one model probably reduced the explanatory power of this model. The lack of available data on the fatty acid profiles of Antarctic krill from the Southern Ocean sector that corresponds to the proposed feeding area of E1 humpback whales is a possible limitation of this study. Filling the gap in knowledge of the diet, feeding behaviour and hence fatty acid composition of Antarctic krill from east Antarctic regions would advance understanding of interannual variability in fatty acid profiles of E1 humpback whales.

## Conclusions

The high degree of variability in the fatty acid profiles of E1 humpback whales observed in this study was unexpected, as these animals are assumed to be high-fidelity Antarctic krill consumers under the classical feeding paradigm. The paradigm was still supported by our results that showed E1 humpback whales feeding at a similar trophic level as zooplankton species, deriving their energy from a diatom-origin diet and with no significant change in the proportion of Antarctic krill consumed by the E1 population over the past 10 years. Our results indicate that both species-specific metabolism and diet have a direct influence upon the observed interannual variability of blubber fatty acid profiles. That is, there is variability in the fatty acid profiles of the whales’ prey, rather than changes in the whales’ diet. The study further demonstrated that fatty acid profiles of E1 humpback whales are sufficiently distinct from year to year that they can be used to distinguish sampling cohorts. Whilst remarkable, it also served to emphasise that interannual variability has to be taken into consideration when assessing long-term trends. Further insight into the role of prey fatty acid profile variability upon E1 humpback whale blubber fatty acid profiles from year to year would be provided through within-season parallel collection of blubber biopsies and Antarctic krill from the corresponding Antarctic feeding ground, the summer preceding migration. Additional insights on the importance of energy acquisition in temperate waters would be gained from higher latitude biopsy collection in late spring as the whales return to the Southern Ocean.

## Materials and methods

### Sample Collection

A total of 348 blubber biopsies were collected from the E1 breeding population between 2008 and 2018 according to methods previously outlined in Waugh et al.^1^, with no sampling conducted in 2010 and 2012. Briefly, the individuals were targeted off North Stradbroke Island, southeast Queensland, Australia (approximately 27°26′S, 153°34′E). Biopsy collection occurred either during the last two weeks of June and the first two weeks of July (northward migration), or the last two weeks of September and the first two weeks of October (southward migration), or during the northward and southward migration each year. Biopsies were obtained with a modified 0.22 calibre rifle (Paxarms NZ) and flotation darts. Biopsy darts were fired at the whale’s dorsum, ventral and slightly posterior to the dorsal fin as recommended by Lambertsen et al.^62^. Blubber biopsies were immediately sub-sectioned, with the lipid fraction sub-sectioned at a blubber core depth of 3–4 cm. Here, only the outer blubber layer is sampled because a study conducted by Waugh et al.^63^ showed that there was no significant difference in lipid and FA profiles between the inner and outer blubber layer in E1 humpback whales. All samples were stored on ice in the field, and then transferred to – 20 °C freezers until lipid and fatty acid analysis. Sub-samples of skin were similarly stored at – 20 °C for genetic sex determination^64^. Observational notes were recorded for pod composition. Females accompanied by a dependent calf were identified as lactating mothers for data interpretation. Sampling was undertaken according to the protocols approved by Griffith University’s Animal Ethics Committee (Griffith University, Ref No: ENV/10/15/AEC) and Moreton Bay marine parks sampling restrictions (Moreton Bay Marine Park Permit #QS2014/CVL1397). All experimental protocols were carried out in accordance with relevant guidelines and regulations.

### Lipid extraction and class determination

Lipids were extracted overnight from pre-weighed (ca. 0.03 g) blubber samples using a modified Bligh and Dyer^65^ method as previously described^1^, with highly purified nanograde solvents. Samples from the years 2008–2013 were lipid extracted using a methanol-chloroform-water (MeOH/CHCl_3_/Milli-Q H_2_O) mixture (2:1:0.8 v/v/v), while samples from 2014 to 2018 were lipid extracted using a methanol-dichloromethane-water (MeOH/CH_2_Cl_2_/Milli-Q H_2_O) mixture (2:1:0.8 v/v/v) due to the increasing awareness of occupational health and safety. The identical efficiency of the two solvent mixtures has been validated^66^. The lower chloroform or dichloromethane phase, containing the total lipids, was collected, reduced to dryness and re-weighed to obtain the total lipid content, expressed as percent lipid of the initial blubber sample. Samples were then re-dissolved in 1.5 ml CH_2_Cl_2_ and an aliquot of 10 mg lipid ml^−1^ CH_2_Cl_2_ of each sample was used to establish the lipid class profile with an Iatroscan MK-5 TLC/FID analyser (Iatron Laboratories, Tokyo, Japan)^67^. The flame ionisation detector was calibrated with a standard solution of known quantities of wax esters (WE), triacylglycerols (TAG), free fatty acids (FFA), sterols (ST), and phospholipids (PL), with hydrocarbon (HC; squalene) also used in a separate standard solution. Aliquots were spotted on chromarods, developed in a solvent system of hexane:diethyl-ether:acetic acid (90:10:0.1 ml) and a dried for 5 min at 50 °C. Results are provided in Fig. S2.

### Fatty acid determination

Fatty acid methyl esters (FAME) were produced by trans-methylation of an aliquot of the total lipid extract with 3 ml of MeOH/HCl/CH_2_Cl_2_ (3 ml 10:1:1 v/v/v) at 100 °C for 1 h. Afterwards, samples were cooled and 1 ml of Milli-Q H_2_O and 2 ml hexane/dichloromethane (4:1 C_6_/CH_2_Cl_2_ v/v) were added, and the phases separated by centrifugation for 5 min at 2000 rpm to obtain the top layer. The lower layer was extracted two more times after the addition of 2 ml 4:1 C_6_/CH_2_Cl_2_ and centrifugation. Before analysing FAME extracts using a gas chromatograph (Agilent Technologies 7890A GC-FID System, Palo Alto, CA, USA) equipped with a non-polar Supelco Equity-1 fused silica capillary column (15 m × 0.1 mm internal diameter, 0.1 μm film thickness)^68^, 1.5 ml of internal injection standard (23:0 FAME) was added. At an oven temperature of 120ºC with helium as the carrier gas, 0.2 µl of samples were injected in splitless mode. The oven temperature was increased by 10 °C every minute until 270 °C, and then by 5 °C every minute until 310 °C. Fatty acid peaks were quantified using the ChemStation software (Agilent Technologies, Rev B.03.01, Palo Alto, CA, USA). Initial identification of fatty acid peaks was based on comparison of retention times with known (Nu Chek Prep mix; https://www.nu-chekprep.com) and a fully characterised laboratory standard (tuna oil). Individual fatty acid peaks are expressed as a percentage of the total fatty acid area.

To confirm FAME identifications, representative samples were analysed on a Thermo Scientific (Waltham, MA, USA) 1310 gas chromatograph-mass spectrometer coupled with a TSQ triple quadruple. A Tripleplus RSH (Waltham, MA, USA) auto sampler was used for sample injection onto a non-polar HP-5 Ultra 2 bonded-phase column (50 m length × 0.32 mm internal diameter × 0.17 µm film thickness). The HP-5 column had a similar polarity as the column used for GC analyses and helium was used as the carrier gas. Initially, the oven temperature of 45 °C was held for 1 min before it was increased by 30 °C every minute until 140ºC, and then by 3 °C every minute until 310 °C, which was held for 12 min. The operating conditions of the mass-spectrometer were the following: electron impact energy 70 eV, emission current 250 µAmp, transfer line 310 °C; source temperature 240 °C; scan rate 0.8 scan/s and mass range 40–650 Da. The mass spectra were processed with the Xcalibur 4.3 software (Thermo Scientific ,Waltham, MA, USA), and peaks were identified and quantified using the same standards as GC-FID analysis. Fatty acids present at < 0.5% were not included in the statistical treatment of the results. According to the fatty acid trophic biomarker concept, ratios of fatty acids can be used as indexes for diet origins. Here we calculated the ratios of 16:1ω7c/16:0 and EPA/DHA as indexes of a diatom-origin diet, and 18:1ω7c/18:1ω9c as a measure to distinguish between a carnivorous and herbivorous diet. Additionally, we calculated the modified carnivory index (CI) introduced by Bode et al.^69^ by dividing the 18:1ω9c content by the sum of all herbivorous biomarkers and 18:1ω9c as follows: CI = 18:1ω9c/(16:1ω7c + 18:1ω7c + 18:4ω3 + 18:1ω9c). The modified CI ranges from 0 for herbivores to 1 for carnivores.

### Environmental data

The most meaningful and readily available parameters associated with Antarctic krill abundance (sea ice concentration; sea surface temperature, SST; chlorophyll *a*, CHL; El-Niño Southern Oscillation Index; ENSO; Southern Annular Mode, SAM) were used as input into a discriminant analysis distance based linear model (DistLM)^70^. Sea-ice extent, duration, thickness, on-set and melt are arguably the most important factors governing Antarctic krill abundance as krill are associated with sea-ice at all life cycle stages^71^. Therefore, sea-ice concentration is commonly used as a measure of the cryosphere in Southern Ocean models^72^. Warming ocean temperatures also directly impact Antarctic krill as they have a narrow temperature optimum, and even changes of 1 to 2ºC could affect the physiological performance, distribution and behaviour of Antarctic krill^71^. Consequently, SST and modes like ENSO, which describes sea surface temperature and atmospheric pressure, or SAM, which describes atmospheric variability, are often used in Southern Ocean models as well^73–75^. Additionally, CHL is commonly used as an overall measure of ecosystem productivity^76^.

**Figure 6:**
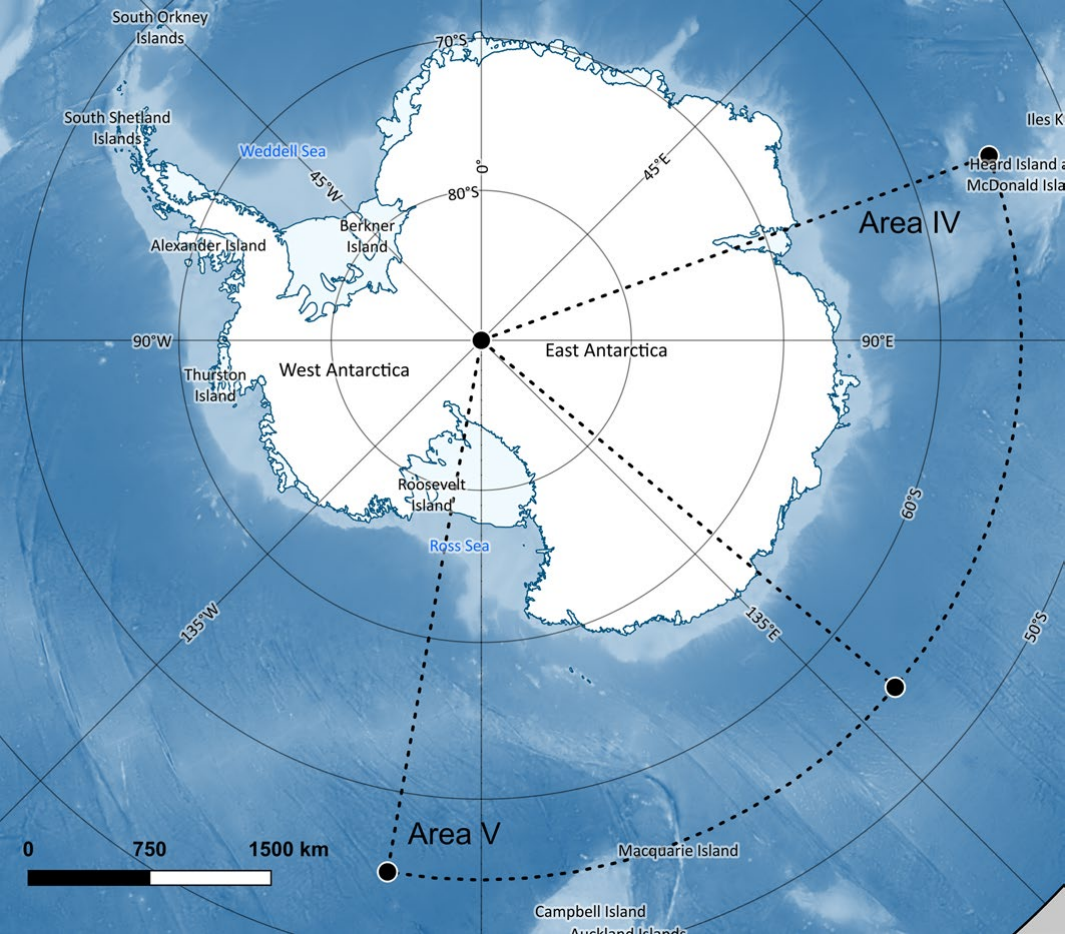
Map of the Southern Ocean showing the location of the IWC Management Areas IV and V, which are the hypothesised feeding areas of the E1 humpback whale breeding population (IWC^10^). Map was produced using the Quantarctica 3 data package (developed by the Norwegian Polar Institute; https://quantarctica.npolar.no) in QGIS (version 3.8; www.qgis.org). Base layers courtesy of the SCAR Antarctic Digital Database.

Average annual austral summer measurements were calculated for the period between the 1st November and the 30th April. Historical SAM data were sourced from the British Antarctic Survey website (https://www.nerc-bas.ac.uk/icd/gjma/sam.html). El-Niño Southern Oscillation Index data were obtained from the National Ocean and Atmospheric Administration (NOAA) website (https://www.esrl.noaa.gov/psd/gcos_wgsp/Timeseries/SOI/). Chlorophyll *a* and SST were sourced from remote sensing data obtained from the National Aeronautics and Space Administration (NASA) website *Oceancolor* (https://oceandata.sci.gsfc.nasa.gov/). The mean monthly CHL concentration and the mean monthly SST were calculated from the Moderate Resolution Imaging Spectroradiometer (MODIS) satellite data. Historical sea ice concentration data were obtained from the French Institute for Exploitation of the Sea website *CERSAT* (https://cersat.ifremer.fr/data/tools-and-services/quicklooks/sea-ice/ssm-i-sea-ice-concentration-maps). Mean monthly sea ice concentrations were calculated from daily satellite data. All calculations were carried out by the authors. Chlorophyll *a*, sea ice concentration and SST data were constrained to the geographical area that corresponds to the IWC feeding area V between 130°E and 170°W, south of 55ºS (Fig. 6). All data were sourced from publicly available websites.

### Data analysis

Lipid content, lipid class and FA percentage data were analysed in R (version 3.5.3) and PRIMER v7 with PERMANOVA + add-on (https://www.primer-e.com). All data were tested for normality using the Shapiro–Wilk test, and homogeneity of variance was tested using Levene’s test. Both assumptions were violated and non-parametric statistical methods were used instead. Results were interpreted with a significance level of α = 0.05. A subset of 272 individuals of known sex (F = 99, M = 173) was incorporated in all analyses, showing that sexes did not significantly vary with respect to total lipid content, lipid classes or fatty acid percentages (Table S6). Therefore, sex was not considered in subsequent analyses. Euclidean distance matrices were calculated for all multivariate statistical analyses. Interannual variation in E1 humpback whale total lipid content was tested using one-factor permutational MANOVA (PERMANOVA) with “year” as a fixed factor and a *post-hoc* pairwise analysis. Apparent temporal variation in the total lipid content was further investigated by comparing the sampled north and south migrating E1 humpback whales separately using a two-factor PERMANOVA. The same PERMANOVA design was also used to test differences among lipid classes. *Post-hoc* pairwise comparisons were used to identify significant differences among factor levels. Fatty acid data were square-root transformed prior to analyses to weight the contributions of common and rare observations and allow representation of fatty acids present in smaller percentages. A two-factor PERMANOVA with “year” and “migration” as fixed factors and “total lipid content” as a covariate was used to test if differences of fatty acid profiles among years and between migrations were significant. Post-hoc pairwise analyses were carried out. Principal component analysis (PCA) was performed to explore differences of fatty acid profiles among years and between migrations, and to identify those fatty acids that explain most of the variability in the data set. Subsequently, a canonical analysis of principal coordinates (CAP) was used to assess how distinct groups of years are from one another based on fatty acid profiles. A DistLM with the “Forward” selection procedure and “adjusted R^2^ “ as the selection criteria was used to identify potential parsimonious models for the fatty acid assemblage of E1 humpback whales in response to five environmental variables and indices. This dissimilarity-based multivariate multiple regression analysis was applied to the full data set using all fatty acids as well as a subset of the data, containing only the major dietary fatty acids^15^. This approach was taken to understand the relation of the diet of this species to environmental variables and indices in more detail. We modelled within-year variation, and variation with a 1-year lag phase and 2-year lag phase.

## Supplementary information

Supplementary Information.

## Data Availability

The datasets generated during and/or analysed during the current study are available from the corresponding author on reasonable request.
