# Supplementary material for: Interannual variability in the lipid and fatty acid profiles of east Australia-migrating humpback whales (Megaptera novaeangliae) across a 10-year timeline
Source: Sci Rep. 2020 Oct 26;10:18274. doi: 10.1038/s41598-020-75370-5 (PMC7589506; doi:10.1038/s41598-020-75370-5)
Supplement: Supplementary file 1 — Supplementary Information. [file 41598_2020_75370_MOESM1_ESM.pdf]

Interannual Variability in the Lipid and Fatty Acid Profiles of East Australia-Migrating Humpback Whales (*Megaptera novaeangliae*) across a 10-year timeline

Jasmin Groß[1,*], Patti Virtue[2,3], Peter D. Nichols[2,3], Pascale Eisenmann[4], Courtney A. Waugh[5], Susan Bengtson Nash[1]

[1] Southern Ocean Persistent Organic Pollutants Program, Environmental Futures Research Institute, Griffith University, 170 Kessels Road, 4111 Nathan, QLD, Australia

[2] Institute for Marine and Antarctic Studies, University of Tasmania, 20 Castray Esplanade, 7004 Hobart, TAS, Australia

[3] CSIRO Oceans and Atmosphere, Castray Esplanade, 7004 Hobart, TAS, Australia

[4] Property NSW – Environmental Science Group, Parramatta Square, Parramatta, NSW, Australia

[5] Animal Science Programme, Nord University, Universitetsalléen 11, 8026 Bodø, Norway

*Correspondence to: *Email address:* J.gross@griffith.edu.au (J. Groß).

ABSTRACT

Southern hemisphere humpback whales are classified as high-fidelity Antarctic krill consumers and as such are vulnerable to variations and long-term changes in krill biomass. Evidence of heterogeneous feeding patterns of east coast of Australia migrating humpback whales has been observed, warranting a comprehensive assessment of interannual variability in their diet. We examined the lipid and fatty acid profiles of east coast of Australia migrating humpback whales sampled between 2008 and 2018. The use of live-sampled blubber biopsies showed that fatty acid profiles varied significantly among all years. The two trophic indicator fatty acids for Antarctic krill, 20:5ω3 and 22:6ω3 remained largely unchanged across the 10-year period, suggesting that Antarctic krill is the principal prey item. A distance-based linear model showed that 33 % of the total variation in fatty acid profiles was explained by environmental variables and climate indices. Most of the variation was explained by the Southern Annular Mode (23.7%). The high degree of variability observed in this study was unexpected for a species that is thought to feed primarily on one prey item. We propose that the observed variability likely arises from changes in the diet of Antarctic krill rather than east coast of Australia migrating humpback whales.

Keywords: Antarctic krill, climate change, diet, migration, sentinel species, southern hemisphere, Southern Ocean

Supplementary Material

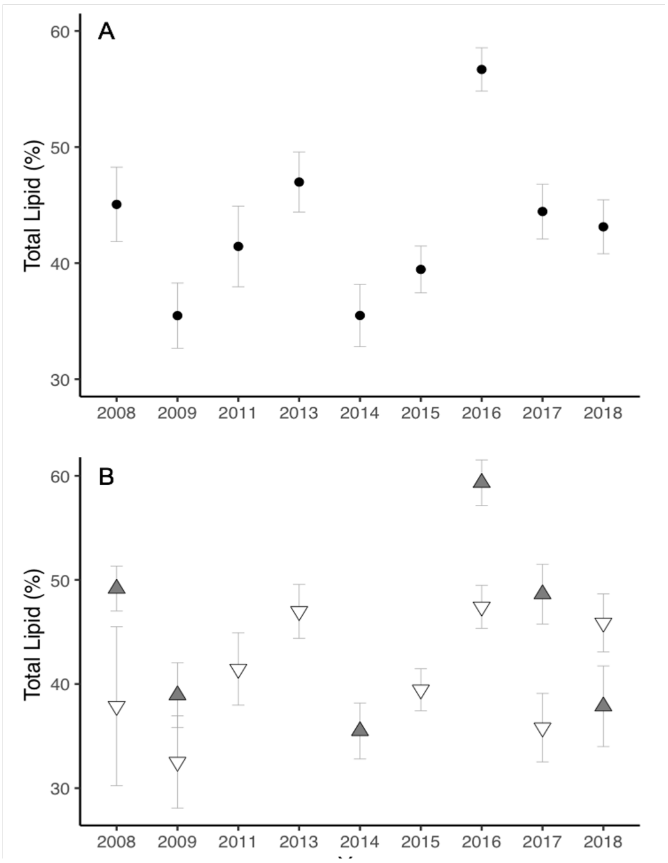

Figure S1. The average total lipid content of E1 humpback whales from 2008 to 2018 (A), separated by the north (grey; B) and south migration (white; B). Error bars represent the standard error of the mean. All samples were included in these graphs (n=348).

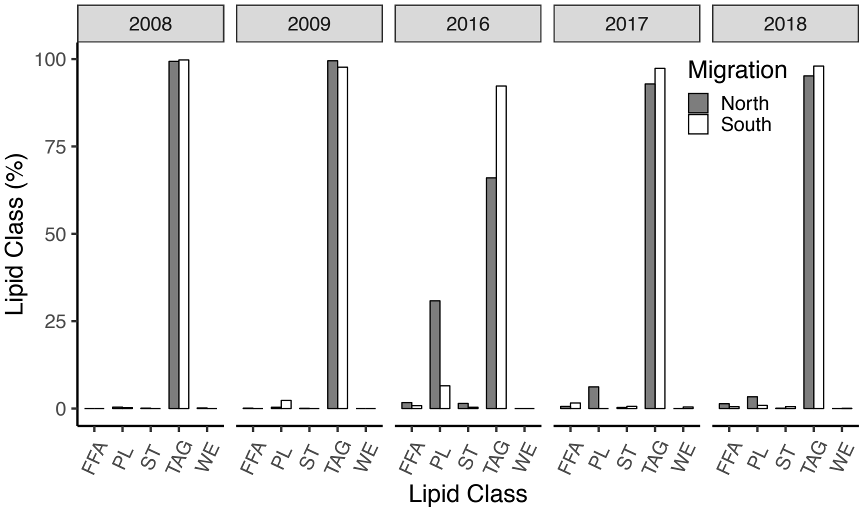

Figure S2. The proportional distribution of lipid classes. Wax esters (WE), triacylglycerols (TAG), free fatty acids (FFA), sterols (ST) and polar phospholipids (PL) are separated by the north- (grey) and south migration (white) of E1 humpback whales for the years 2008, 2009, 2016 and 2017 (n=142).

Table S1. PERMANOVA Pairwise Comparison of total lipid content of E1 humpback whales among years within level migration (Group). Table shows the t value (t), the p value using a significance level of α = 0.05 and the number of permutations. * indicates significant differences (n-194).

Group	Pairwise Comparison	t	P-value	Permutations
North Migration	2008 - 2009	2.76	0.0103*	9841
	2008 - 2014	2.95	0.0041*	9833
	2008 - 2016	2.37	0.0208*	9837
	2008 - 2017	0.11	0.91	9837
	2008 - 2018	2.65	0.0121*	9846
	2009 - 2014	0.67	0.51	9832
	2009 - 2016	4.33	0.0001*	9822
	2009 - 2017	1.89	0.0667*	9818
	2009 - 2018	0.22	0.83	9798
	2014 - 2016	6.94	0.0001*	9812
	2014 - 2017	3.34	0.0019*	9842
	2014 - 2018	0.45	0.65	9833
	2016 - 2017	3.00	0.004*	9821
	2016 - 2018	4.44	0.0003*	9835
	2017 - 2018	2.03	0.05*	9851
South Migration	2008 - 2009	0.65	0.52	9574
	2008 - 2011	0.47	0.65	9830
	2008 - 2013	1.43	0.16	9831
	2008 - 2015	0.25	0.80	9840
	2008 - 2016	1.52	0.14	9688
	2008 - 2017	0.29	0.77	9741
	2008 - 2018	1.24	0.23	9816
	2009 - 2011	1.55	0.14	9814
	2009 - 2013	2.96	0.005*	9840
	2009 - 2015	1.45	0.15	9822
	2009 - 2016	3.05	0.005*	9849
	2009 - 2017	0.61	0.55	9842
	2009 - 2018	2.69	0.01*	9836
	2011 - 2013	1.31	0.20	9835
	2011 - 2015	0.51	0.60	9829
	2011 - 2016	1.20	0.24	9839
	2011 - 2017	1.10	0.28	9852
	2011 - 2018	0.98	0.34	9814
	2013 - 2015	2.22	0.03*	9830
	2013 - 2016	0.10	0.92	9860
	2013 - 2017	2.58	0.01*	9829
	2013 - 2018	0.29	0.78	9831
	2015 - 2016	1.80	0.08*	9842
	2015 - 2017	0.84	0.40	9851
	2015 - 2018	1.71	0.09	9814
	2016 - 2017	2.89	0.01*	9816
	2016 - 2018	0.39	0.70	9844
	2017 - 2018	2.33	0.03*	9836

Table S2. PCA Eigenvalue Scores of fatty acid percentages from 2008 to 2018 for the 17 most abundant fatty acids of E1 humpback whales (n=348).

Fatty Acid	PC1	PC2	PC3	PC4	PC5
14:00	0.047	-0.013	0.131	-0.706	0.204
16:00	0.164	-0.053	0.136	-0.364	0.469
18:00	0.086	-0.047	0.047	0.093	0.217
14:01	0.067	0.112	0.013	-0.493	-0.734
16:1ω7c	-0.075	-0.143	0.715	0.043	-0.158
18:1ω9c	-0.024	-0.275	0.59	0.11	0.037
18:1ω7c	-0.054	0.019	-0.058	-0.08	0.188
20:1ω9	-0.05	-0.052	-0.009	-0.156	0.136
18:4ω3	-0.181	0.08	0.015	-0.13	0.028
18:2ω6	-0.193	0.149	0.123	0.101	-0.187
18:3ω3	-0.096	0.028	0.02	0.036	-0.028
20:4ω6	-0.124	-0.001	-0.017	0.064	-0.017
20:5ω3	-0.591	0.253	0.055	-0.042	0.064
20:4ω3	-0.158	0.054	0.021	-0.004	0.006
21:5ω3	0	0.656	0.226	0.112	0.141
22:6ω3	-0.481	0.19	-0.02	-0.154	0.111
22:5ω3	-0.497	-0.567	-0.168	-0.039	-0.012

Table S4. CAP Cross Validation results based on fatty acid percentages from 2008 to 2018 for the 17 most abundant fatty acids of E1 humpback whales. Table shows the actual sampling year and the number of samples assigned to each year based on fatty acid profiles as well as the percentage of correctly assigned samples (% Correct; n=345).

Sampling Year	2008	2009	2011	2013	2014	2015	2016	2017	2018	Total	% Correct
2008	11	6	1	0	1	0	0	0	0	19	57.9
2009	0	23	2	0	0	0	0	0	0	25	92.0
2011	2	1	23	0	0	0	0	0	0	26	88.5
2013	0	0	0	30	1	0	0	0	0	31	96.8
2014	0	0	1	0	18	10	3	0	0	33	54.6
2015	0	0	0	1	4	58	1	0	0	64	90.6
2016	0	0	0	2	1	25	35	0	0	63	55.6
2017	0	0	0	0	0	0	1	48	0	49	98.0
2018	0	0	2	1	2	3	1	0	26	35	74.3

Table S5. PCA Eigenvalue Scores of fatty acid percentages of E1 humpback whales from 2008 to 2018 and possible prey species. Results are based on 10 fatty acids that were reported in all studies (n=348).

Fatty Acid	PC1	PC2	PC3	PC4	PC5
14:00	0.067	-0.208	0.425	-0.207	0.015
16:00	-0.176	-0.456	0.673	-0.158	0.178
18:00	0.018	-0.038	0.116	0.069	0.054
16:1ω7c	0.494	-0.32	-0.295	-0.625	-0.185
18:1ω9c	0.646	-0.364	0.006	0.634	0.188
18:1ω7c	0.157	-0.185	0.006	-0.155	-0.186
20:1ω9	0.051	0.12	-0.125	-0.247	0.803
18:2ω6	0.055	-0.003	-0.151	-0.138	0.442
20:5ω3	-0.366	-0.546	-0.398	0.021	-0.041
22:6ω3	-0.372	-0.407	-0.262	0.171	0.139

Table S3. PERMANOVA Pairwise Comparison of fatty acid percentages of E1 humpback whales between years within level "migration" (Group) and between migrations within level "year". Table shows the t value (t), the p value using a significance level of α = 0.05 and the number of permutations. * indicates significant differences (n=194).

Group	Pairwise Comparison	t	P-value	Permutations
North Migration	2008 - 2009	2.0254	0.0455	9920
	2008 - 2014	3.5737	0.0001	9946
	2008 - 2016	3.9324	0.0001	9947
	2008 - 2017	5.64	0.0001	9935
	2008 - 2018	2.8834	0.0023	9942
	2009 - 2014	3.9152	0.0001	9942
	2009 - 2016	3.5382	0.0001	9942
	2009 - 2017	7.819	0.0001	9938
	2009 - 2018	4.2561	0.0001	9908
	2014 - 2016	2.4407	0.0026	9924
	2014 - 2017	8.7281	0.0001	9941
	2014 - 2018	3.4811	0.0001	9941
	2016 - 2017	6.5285	0.0001	9946
	2016 - 2018	2.7066	0.0008	9928
	2017 - 2018	7.9298	0.0001	9942
South Migration	2008 - 2009	2.2067	0.0128	8729
	2008 - 2011	2.0627	0.0083	9890
	2008 - 2013	3.3192	0.0006	9908
	2008 - 2015	5.8606	0.0001	9901
	2008 - 2016	3.1018	0.0001	8794
	2008 - 2017	3.5139	0.0001	9290
	2008 - 2018	3.2009	0.0002	9843
	2009 - 2011	2.2195	0.001	9932
	2009 - 2013	4.6869	0.0001	9942
	2009 - 2015	7.3908	0.0001	9948
	2009 - 2016	4.2892	0.0001	9952
	2009 - 2017	6.2997	0.0001	9922
	2009 - 2018	4.4516	0.0001	9940
	2011 - 2013	4.0958	0.0001	9943
	2011 - 2015	6.3657	0.0001	9931
	2011 - 2016	3.3391	0.0001	9956
	2011 - 2017	6.6051	0.0001	9947
	2011 - 2018	4.5502	0.0001	9939
	2013 - 2015	3.6914	0.0001	9949
	2013 - 2016	2.9897	0.0001	9955
	2013 - 2017	6.8653	0.0001	9944
	2013 - 2018	5.5181	0.0001	9948
	2015 - 2016	2.3941	0.0008	9944
	2015 - 2017	11.731	0.0001	9936
	2015 - 2018	7.9217	0.0001	9939
	2016 - 2017	6.2304	0.0001	9941
	2016 - 2018	4.2619	0.0001	9938
	2017 - 2018	7.2845	0.0001	9939
2008	North - South	2.1545	0.032*	977
2009	North - South	1.8559	0.009*	998
2016	North - South	2.3276	0.01*	999
2017	North - South	1.1574	0.234	998
2018	North - South	0.67024	0.681	997

Table S6. Kruskal-Wallis rank sum test with sex as the independent variable and total lipid content, fatty acid classes and individual fatty acids. Table shows the *Chi*-square value (X^2), the degrees of freedom (df) and p value using a significance level of α = 0.05. * (n=272).

Dependent Variable	*Chi* - value	df	*p* - value
Total Lipid Content	0.017	1	0.8977
Fatty Acid Classes	0.143	1	0.7054
Fatty Acids	0.238	1	0.6255
